# Role of γδ T cells in turkey herpesvirus vaccine protection against Marek’s disease virus

**DOI:** 10.1099/jgv.0.002204

**Published:** 2026-01-30

**Authors:** Mohammad A. Sabsabi, Ahmed Kheimar, Dominik von La Roche, Sonja Härtle, Dusan Kunec, Yulin Cong, Lisa Kossak, Theresa von Heyl, Benjamin Schusser, Benedikt B. Kaufer

**Affiliations:** 1 Institut für Virologie, Freie Universität Berlin, Berlin 14163, Germany; 2 Veterinary Centre for Resistance Research (TZR), Freie Universität Berlin, Berlin 14163, Germany; 3 Department of Poultry Diseases, Faculty of Veterinary Medicine, Sohag University, Sohag 82524, Egypt; 4 Department of Veterinary Sciences, Ludwig-Maximilians-Universität München, Planegg 82152, Germany; 5 Reproductive Biotechnology, TUM School of Life Sciences, Technische Universität München, Freising 85354, Germany; 6 Center for Infection Prevention (ZIP), Technische Universität München, Freising 85354, Germany

**Keywords:** γδ T cells, lymphoma, Marek's disease virus (MDV), oncogenesis, turkey herpesvirus (HVT)

## Abstract

γδ T cells are a highly abundant lymphocyte subset in chickens and play key roles in early immune responses to infection. It has been recently shown that γδ T cells restrict Marek’s disease virus (MDV) pathogenesis; however, it remained elusive if they play a role in vaccine protection. In this study, we vaccinated γδ T-cell-knockout chickens with the commercial turkey herpesvirus (HVT) vaccine and challenged them with very virulent MDV. The disease incidence was significantly increased in vaccinated chickens in the absence of γδ T cells. This increase was comparable to a previous study in unvaccinated γδ T-cell-knockout chickens, suggesting that γδ T cells only play a minor role in vaccine protection. Furthermore, the viral load in the spleen was significantly increased in the absence of γδ T cells. Interestingly, viral load in the skin and in dust shed by the animals was drastically increased, suggesting that the absence of γδ T cells affects MDV shedding. In addition, we quantified various immune cell subsets to determine if these could be responsible for the observed phenotypes. Together, our data indicate that γδ T cells only play a minor role in HVT-mediated protection, but their absence drastically affects shedding of this deadly pathogen in vaccinated animals.

## Introduction

Marek’s disease virus (MDV) is an alphaherpesvirus that causes one of the most prevalent virus-induced cancers in the animal kingdom [1]. The virus causes immunosuppression, neurological disorders and fatal T-cell lymphomas, as well as a mortality of up to 100% in unvaccinated chickens [2]. MDV infects its host primarily via the inhalation of contaminated dust. Upon inhalation, the virus is transported to the lymphoid organs, where it infects B and T cells. The virus spreads within the host in a cell-associated manner and establishes latency primarily in (Cluster of Differentiation 4) CD4^+^ αβ T cells. These cells can also be transformed, resulting in deadly lymphomas [3]. In addition, these cells can transport the virus to the feather follicle epithelium (FFE), where cell-free virus is produced and shed into the environment with the dander [4].

To protect chickens against MDV, billions of animals are vaccinated with live (attenuated) vaccines every year [5]. Three commercial vaccines are widely used: (i) the turkey herpesvirus [HVT; *Mardivirus meleagridalpha1* (MeAHV1)], (ii) the naturally apathogenic *Mardivirus gallidalpha3* (GaAHV3) strain SB-1 and (iii) the attenuated GaAHV2 strain CVI988/Rispens [6,8]. HVT is also extensively used as a vector vaccine, providing protection against other pathogens such as infectious bursal disease virus, Newcastle disease virus, avian influenza virus and infectious laryngotracheitis virus [9,11], aside from protecting against MDV. Even though these vaccines have been used for decades, vaccine protection and the contribution of specific immune cell subsets remain poorly understood.

γδ T cells are highly abundant T cells in chickens and are thought to contribute to the early immune defence and long-term immunity [5,12]. γδ T cells can react quickly to infections, secrete cytokines and regulate immune responses [13]. We recently demonstrated that γδ T cells play an important role in MDV pathogenesis, as disease and tumour incidence drastically increased in their absence. γδ T-cell-knockout chickens exhibited higher viral levels in the thymus and spleen, suggesting that γδ T cells restrict MDV replication in these organs [14]. Upon vaccination, γδ T cells drastically expand in multiple organs and show an increased cytotoxic activity [15,16], suggesting that these cells may have a role in vaccine-induced immunity. However, their precise role in vaccine protection remained unclear.

To assess the role of γδ T cells in vaccine protection, we vaccinated γδ T-cell-knockout chickens and challenged them with a very virulent MDV. Our data revealed that disease incidence was significantly increased in the absence of γδ T cells. This was comparable with the increase in unvaccinated γδ T-cell-knockout chickens [14], suggesting that γδ T cells only play a minor role in HVT-induced vaccine protection. Consistently, virus levels were significantly increased in the spleen. In addition, virus load was also drastically increased in the skin and in the dust, suggesting that the absence of γδ T cells impacts shedding of this highly oncogenic herpesvirus.

## Methods

### Cells and viruses

Chicken embryo cells were generated from specific pathogen-free VALO eggs (VALO BioMedia GmbH, Osterholz-Scharmbeck, Germany) as described previously [17]. The cells were then cultured in Eagle’s minimal essential medium (PAN Biotech, Aidenbach, Germany), supplemented with 1–10% FBS (PAN Biotech) and 1% penicillin (100 U ml^−1^)/streptomycin (100 µg ml^−1^) (AppliChem, Darmstadt, Germany). Cells were cultured in a humidified incubator at 37 °C and 5% Carbon dioxide (CO₂). The chickens were vaccinated using the HVT FC-126 vaccine strain. The very virulent RB-1B strain was used as a challenge virus, as described previously [14]. The viral stocks were subsequently frozen, stored in liquid nitrogen and titrated prior to their use [18].

### Animals and genotyping

To investigate the role of γδ T cells, we used genetically modified γδ T-cell-knockout chickens (TCR Cγ^−/−^) that have been thoroughly characterized previously [19]. Genotyping was carried out as described [14]. Briefly, peripheral whole blood samples were collected after hatch, and DNA was extracted using the NucleoSpin 96 Blood core kit (Macherey-Nagel, Düren, Germany) according to the manufacturer’s instructions. PCR-based genotyping was conducted usingT cell receptor (TCR)-specific primers following the established protocol [14,19].

### *In vivo* experiment

One-day-old chicks were genotyped and divided into two groups: wild-type (WT; *n*=24) and γδ T-cell-knockout (TCR Cγ^−/−^; *n*=23). Each group was housed in isolation rooms in a Biosafety Level 2 (BSL2) facility with free access to food and water. The chicks were vaccinated subcutaneously with 2,000 p.f.u. of HVT. Five days post-vaccination (dpv), they were challenged subcutaneously with 2,000 p.f.u. of the very virulent RB-1B strain. Both vaccine and virus inoculum were back-titrated after the injection of the animals, which confirmed the intended dose. During the first weeks of life, the chicks had a mild diarrhoea, and we detected *Clostridium perfringens* in the faeces. Peripheral whole blood samples were collected at 7, 10, 14, 21 and 28 dpv. Additionally, feathers were collected from the chickens to quantify virus load in the FFE at 21, 28, 35 and 42 dpv. Dust samples were taken from the air filters in each room at indicated time points to assess virus shedding into the environment. During the experiment, the chickens were monitored twice daily for clinical symptoms caused by MDV. When symptoms were evident or at the end of the experiment (at 90 dpv), the chickens were humanely euthanized and assessed for gross tumour lesions, and spleens were harvested for quantitative PCR (qPCR).

### Quantification of virus genome copies

DNA from blood was extracted using the NucleoSpin 96 Blood Core Kit (Macherey-Nagel, Düren, Germany) following the manufacturer’s instructions. To assess virus shedding, DNA from feather FFE and dust samples was extracted by treatment with proteinase K at 55 °C overnight, followed by phenol:chloroform extraction as described previously [20]. DNA from the spleen was processed using the InnuPREP DNA Mini Kit (Analytik-Jena, Berlin, Germany) according to the manufacturer’s instructions. Vaccination was confirmed by qPCR detection of the HVT SORF1 gene in blood at 7 dpv [21]. Virus genome copies were measured by qPCR using primers and probes specific for infected cell polypeptide (ICP4) and cellular inducible nitric oxide synthase (iNOS), as published previously [22,23]. MDV DNA genome copies were normalized against the iNOS gene [24].

### Flow cytometry

To evaluate the effects of vaccination/infection on various immune cell subsets, absolute cell counts were performed to quantify B cells, CD8^−^ αβ, CD8^+^ αβ and γδ T cells in the blood, as described previously [14]. Briefly, peripheral whole blood samples were collected from the wing veins, stabilized using the TransFix^®^ reagent (Cytomark, Buckingham, UK) according to the manufacturer’s guidelines, diluted in flow buffer and incubated with the published antibody mix [14]. The samples were assessed using a FACSCanto II (Becton Dickinson, Heidelberg, Germany). The data were analysed using FACSDiva (Becton Dickinson, Heidelberg, Germany) and FlowJo_v10.10.0 (FlowJo LLC, Oregon, USA) software [25].

### Statistical analysis

Statistical analyses were conducted using GraphPad Prism version 9 (GraphPad Software, Inc., San Diego, CA, USA). Details of the employed statistical tests are provided in the respective figure legends.

## Results

### HVT-induced vaccine protection in the absence of γδ T cells

**Fig. 1.**
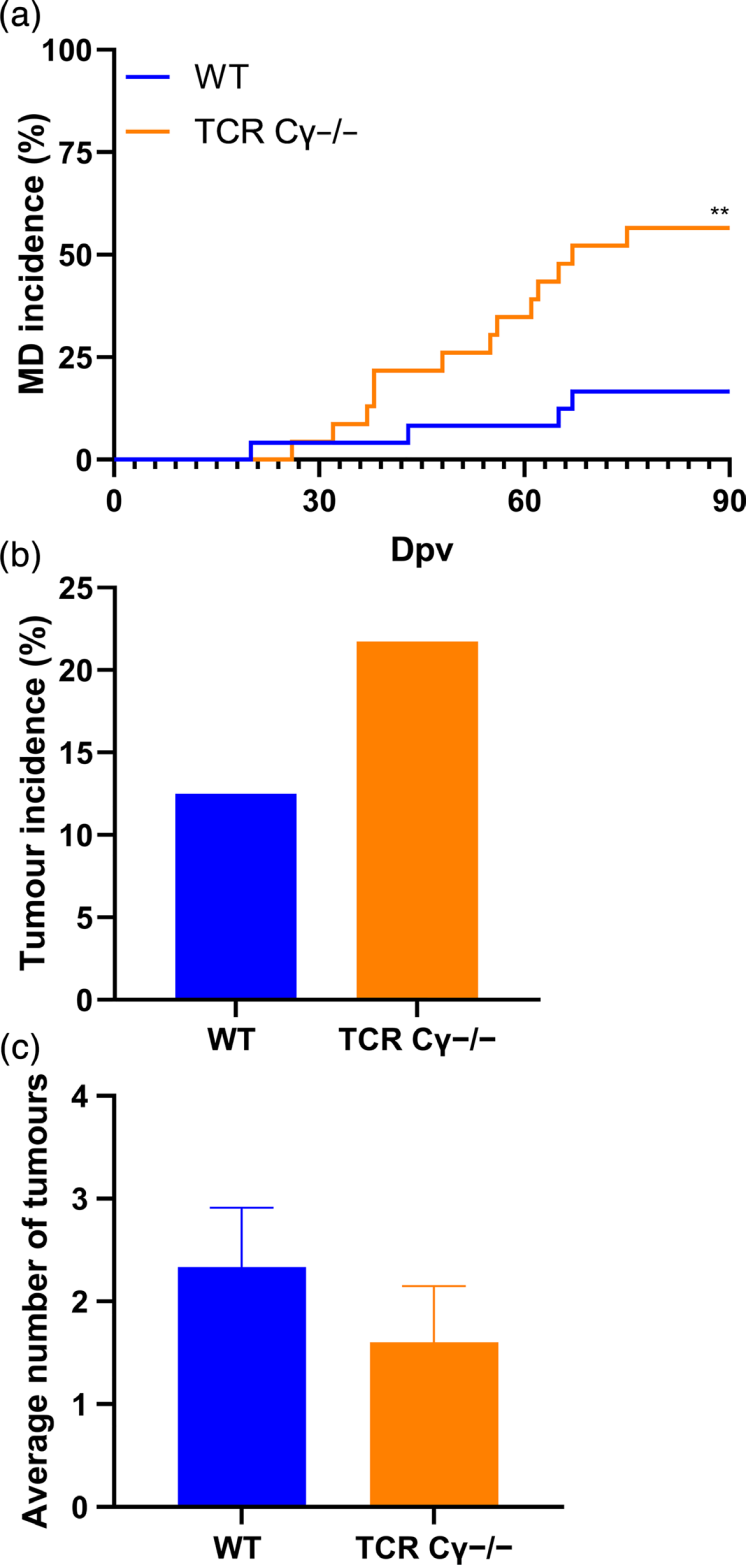
The absence of γδ T cells impairs HVT-induced vaccine protection. (**a**) Marek’s disease incidence in chickens vaccinated with HVT and challenged with very virulent RB-1B (WT; *n*=24; TCR Cγ^−/−^, *n*=23) (Mantel–Cox analysis; **P≤0.01). (**b**) Tumour incidence is shown as the percentage of animals that developed gross tumours (*P*>0.05, Fisher’s exact test). (**c**) Average number of visceral organs with gross tumours in tumour-bearing chickens (*P*>0.05, Fisher’s exact test).

A recent study revealed that the absence of γδ T cells in MDV-infected animals significantly increased disease and tumour incidence [14]. To investigate whether γδ T cells also play a role in vaccine protection, we vaccinated both WT and γδ T-cell-knockout chickens with the commercial HVT vaccine and challenged them with the very virulent RB-1B strain. qPCR analysis confirmed successful vaccination, with all animals (eight out of eight per group) testing positive for HVT in blood samples at 7 dpv. γδ T-cell-knockout chickens had a significantly higher Marek”s disease incidence (56%) compared with their WT counterparts (16%) (Fig. 1a). Tumour incidence was also mildly increased in the absence of γδ T cells (21%) compared with WT counterparts (12%) (Fig. 1b). However, the average number of tumours per bird (Fig. 1c) remained comparable between the two groups. The size and appearance of the tumours were also comparable between both groups. Importantly, a similar increase in disease and tumour incidence was previously observed upon infection of unvaccinated γδ T-cell-knockout chickens [14], indicating that γδ T cells play no, or only a minor, role in HVT vaccine protection.

### Absence of γδ T cells affects MDV replication in HVT-vaccinated chickens

**Fig. 2.**
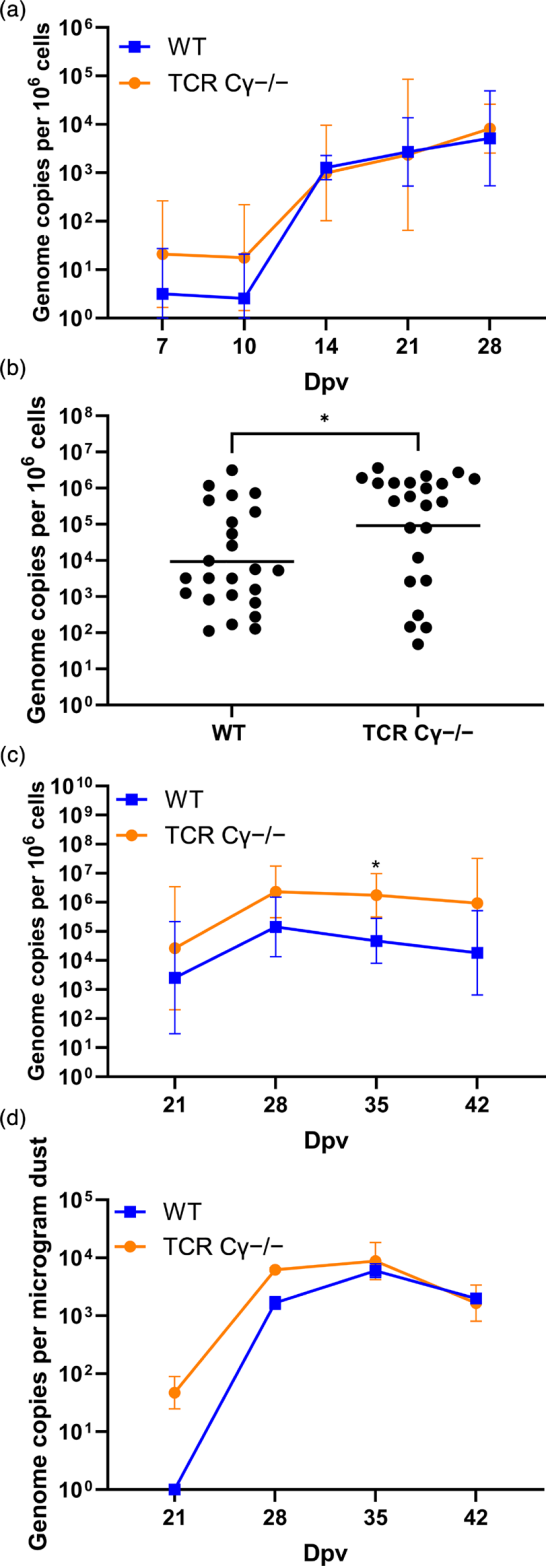
The absence of γδ T cells in HVT-vaccinated chickens affects MDV replication. (a) qPCR analysis of MDV genome copies in the blood of vaccinated/challenged chickens (*n*=8 per group). Data are shown as means±sd (*P*>0.05, Mann–Whitney U test). (**b**) MDV load in spleen tissues of WT and γδ T-cell-knockout (TCR Cγ^−/−^) chickens. The viral load for the individual birds and the mean (line) are shown (*P≤0.05, Mann–Whitney U test). (**c**) qPCR analysis of virus load in the FFE (*n*=8 per group). Data are shown as means±sd (*P≤0.05, Mann–Whitney U test). (**d**) MDV load per 1 µg of dust collected from the rooms of the respective groups at the indicated time points. Data are shown as means±sd (*P*>0.05, Mann–Whitney U test).

To determine if the absence of γδ T cells affects virus levels in vaccinated animals, we assessed the viral load in the blood, spleen, skin and dust. Virus load in the blood was not affected in vaccinated γδ T-cell-knockout chickens compared with their WT counterparts (Fig. 2a). In contrast, MDV load was significantly higher in the spleen in the absence of γδ T cells (Fig. 2b), indicating that γδ T cells may restrict MDV replication in this organ. This is consistent with the increased virus levels in the spleen of unvaccinated γδ T-cell-knockout chickens [14]. Intriguingly, MDV replication was significantly increased by up to 50-fold in the FFE in the absence of γδ T cells (Fig. 2c). This was consistent with a reduction in virus levels in the dust at 21 and 28 dpv (Fig. 2d). A mild increase in the number of HVT copies was also observed in the FFE in the absence of γδ T cells (Fig. S1, available in the online Supplementary Material). Strikingly, an increase in MDV shedding was not observed in unvaccinated γδ T-cell-knockout chickens [14], suggesting that this increase is vaccination dependent and reflects a loss of HVT-induced control in the absence of γδ T cells.

### Effect on immune cell populations in the blood

**Fig. 3.**
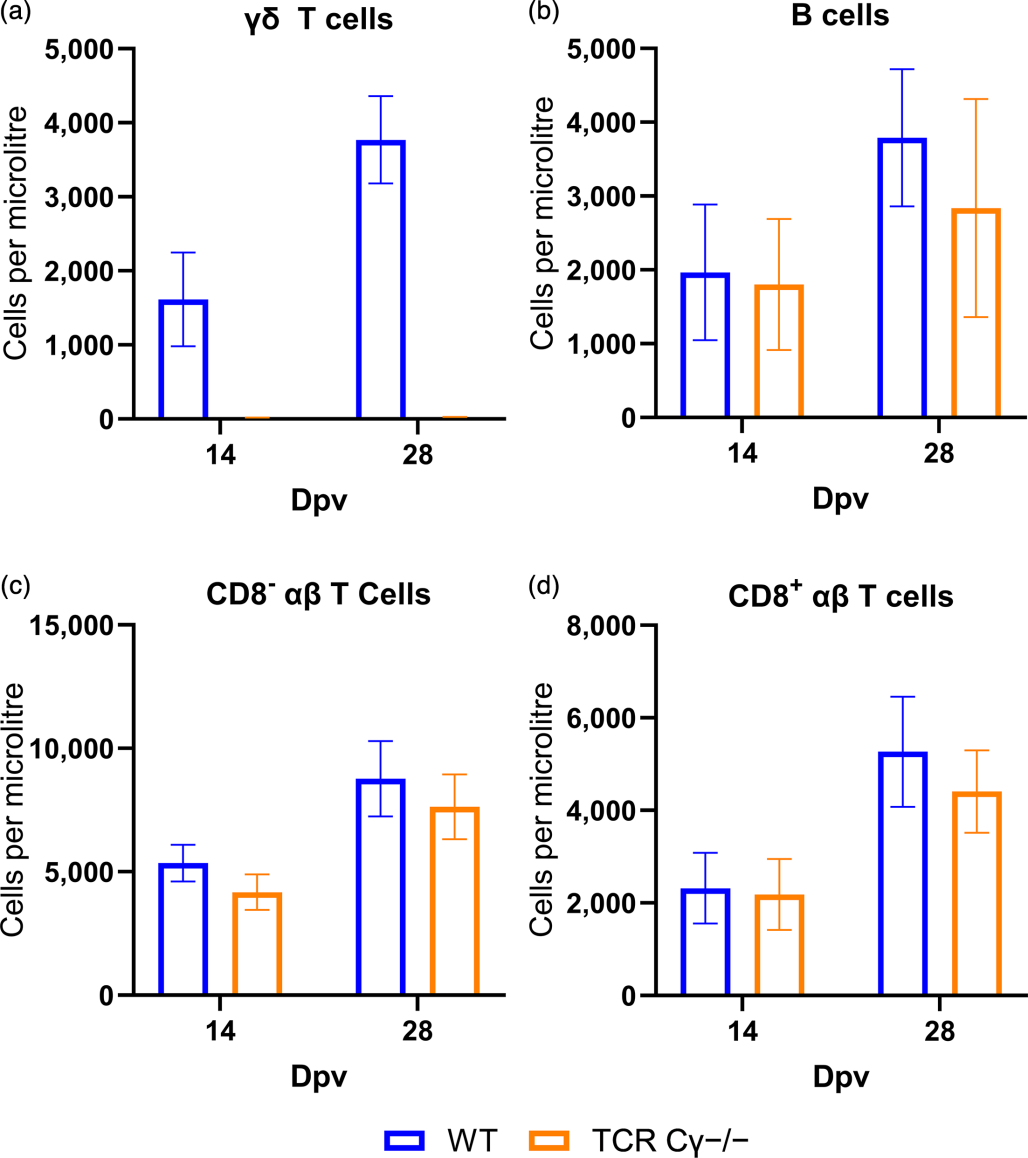
Immune cell populations in the blood. Flow cytometric analysis of immune cells in the peripheral blood at 14 and 28 dpv, γδ T cells (**a**), B cells (**b**), CD8^−^ αβ T cells (**c**) and CD8^+^ αβ T cells (**d**) count in the blood of WT (*n*=8 per group) and TCR Cγ^−/−^ (*n*=8) chickens (*P*>0.05, Mann–Whitney U test).

To determine if other immune cell populations are affected by the absence of γδ T cells in HVT-vaccinated chickens, we examined various immune cell subsets in the blood at 14 and 28 dpv. Flow cytometry confirmed that all γδ T-cell-knockout chickens completely lacked γδ T cells (Fig. 3a), while normal levels were detected in WT animals. This analysis also revealed that the number of B cells was not significantly altered at 14 dpv, and only a minor decrease was observed at 28 dpv (Fig. 3b) in the absence of γδ T cells. Similarly, the absolute number of CD8^−^ and CD8^+^ αβ T cells was not significantly altered in HVT-vaccinated γδ T-cell-knockout chickens (Fig. 3c, d). The results show that the absence of γδ T cells only has minimal impact on these immune cell subsets, suggesting that they are not responsible for the observed phenotype.

## Discussion

γδ T cells are highly abundant T cells in chickens and are thought to play a critical role in the immune response [13]. Upon MDV infection and vaccination, γδ T cells expand and express various cytokines [15,16, 26]. In the absence of γδ T cells, disease and tumour incidence were significantly increased, and higher viral loads were detected in the thymus and spleen, indicating a role for these cells in the defence against MDV [14]. However, the role of γδ T cells in vaccine-induced protection against MDV remained poorly understood. To close this knowledge gap, we vaccinated γδ T-cell-knockout chickens with HVT and challenged them with very virulent MDV. HVT was selected as it is used as a vector vaccine in billions of chickens to protect them against MDV and other important pathogens [9]. In addition, HVT was chosen as the genetic background of the γδ T-cell-knockout chickens (LSL line) is rather resistant against MDV [14], and HVT was shown to provide only partial protection against very virulent MDV strains [27].

Our vaccine/challenge experiments revealed that γδ T-cell-knockout chickens had a significantly higher disease incidence (56%) compared with the WT cohort (17%). Interestingly, a comparable increase in disease incidence was previously observed in unvaccinated γδ T-cell-knockout chickens (70%) versus their WT counterparts (45%) [14]. This not only highlights the partial protection provided by HVT against very virulent MDV but also suggests that γδ T cells play no, or only a minor, role in the protection provided by this MDV vaccine. However, this effect may vary with other commonly used vaccines, such as SB-1 and CVI988, which should be investigated in future studies. The difference in tumour incidence was less pronounced in HVT-vaccinated γδ T-cell-knockout chickens (21%) compared with WT chickens (12%), when compared with experiments involving unvaccinated γδ T-cell-knockout chickens (45%), where a more than twofold increase was observed compared with WT chickens [14]. This could be due to γδ T-cell-independent protective effects induced by the HVT. Overall, these data indicate that γδ T cells do not play a major role in HVT-induced immune protection against disease and tumour formation.

Despite the significantly higher disease incidence observed in the absence of γδ T cells, viral load in the blood remained comparable between γδ T-cell-knockout and WT chickens. In contrast, a significant increase in viral load in the spleen was observed in the absence of γδ T cells. This is consistent with the increased load in the spleen in unvaccinated γδ T-cell-knockout chickens observed previously [14]. This provides further support for a tissue-specific role of γδ T cells in limiting MDV replication. Previous studies also predicted a role of γδ T cells based on their increase in lung and lymphoid tissues and an increase in cytokines at early time points after MDV vaccination [15,16]. This tissue-specific reduction in MDV replication could also be responsible for the increase in Marek’s disease incidence observed in the γδ T-cell-knockout chickens.

Intriguingly, MDV load was significantly higher in the FFE and in dust from HVT-vaccinated animals in the absence of γδ T cells. HVT vaccination has previously been shown to significantly reduce MDV shedding [28]. Despite HVT vaccination, γδ T-cell-knockout chickens shed MDV at levels similar to those in both unvaccinated animal groups in previous studies (1.6×10⁶ versus 1.5×10⁶ copies per microgram dust) [14]. These results suggest that HVT vaccination did not restrict MDV replication in the FFE and shedding in the absence of γδ T cells. Intriguingly, HVT levels in the FFE were also increased in vaccinated chickens in the absence of γδ T cells, indicating that γδ T cells may play a role in reducing virus transmission of both MDV and HVT. Furthermore, analysis of peripheral blood lymphocytes revealed no significant differences in the frequency of B cells or CD8^−^, CD8^+^ αβ T cells between groups. This suggests that the elevated viral load observed in the FFE is specifically linked to the lack of γδ T cells rather than broader changes in immune cell composition.

In summary, our data reveal that γδ T cells play no, or only a minor, role in HVT-induced vaccine protection against MDV. However, the absence of γδ T cells significantly increased virus levels in the FFE and dust, indicating that γδ T cells limit viral replication in the skin and shedding of this deadly pathogen.

## Supplementary material

10.1099/jgv.0.002204 Uncited Fig. S1.

## Abbreviations

dpv: days post-vaccination
FFE: feather follicle epithelia
HVT: turkey herpesvirus
MDV: Marek’s disease virus
qPCR: quantitative PCR
WT: wild-type

## References

[1] Parcells MS, Burnside J, Morgan RW, Robertson ES (2012). Cancer Associated Viruses.

[2] Nair V (2005). Evolution of Marek’s disease -- a paradigm for incessant race between the pathogen and the host. Vet J.

[3] Schat KA, Baranowski E (2007). Animal vaccination and the evolution of viral pathogens. *Rev Sci Tech*.

[4] Davison F, Nair V (2004). Marek’s Disease: An Evolving Problem.

[5] Witter RL (1997). Increased virulence of Marek’s disease virus field isolates. Avian Dis.

[6] Bertzbach LD, Conradie AM, You Y, Kaufer BB (2020). Latest insights into Marek’s disease virus pathogenesis and tumorigenesis. Cancers (Basel).

[7] Jarosinski KW, Tischer BK, Trapp S, Osterrieder N (2006). Marek’s disease virus: lytic replication, oncogenesis and control. Expert Rev Vaccines.

[8] Gimeno IM (2008). Marek’s disease vaccines: a solution for today but a worry for tomorrow?. Vaccine.

[9] Dunn JR, Dimitrov KM, Miller PJ, Garcia M, Turner-Alston K (2019). Evaluation of protective efficacy when combining Turkey herpesvirus–vector vaccines. Avian Diseases.

[10] Esaki M, Noland L, Eddins T, Godoy A, Saeki S (2013). Safety and efficacy of a turkey herpesvirus vector laryngotracheitis vaccine for chickens. Avian Dis.

[11] Esaki M, Godoy A, Rosenberger JK, Rosenberger SC, Gardin Y (2013). Protection and antibody response caused by Turkey herpesvirus vector newcastle disease vaccine. Avian Dis.

[12] Chang S, Xie Q, Dunn JR, Ernst CW, Song J (2014). Host genetic resistance to Marek’s disease sustains protective efficacy of herpesvirus of Turkey in both experimental and commercial lines of chickens. Vaccine.

[13] Fenzl L, Göbel TW, Neulen M-L (2017). Gammadelta T cells represent a major spontaneously cytotoxic cell population in the chicken. Dev Comp Immunol.

[14] Sabsabi MA, Kheimar A, You Y, von La Roche D, Härtle S (2024). Unraveling the role of gammadelta T cells in the pathogenesis of an oncogenic avian herpesvirus. mBio.

[15] Hao X, Li S, Li J, Yang Y, Qin A (2021). An anti-tumor vaccine against Marek’s disease virus induces differential activation and memory response of gammadelta T cells and CD8 T cells in chickens. Front Immunol.

[16] Matsuyama-Kato A, Iseki H, Boodhoo N, Bavananthasivam J, Alqazlan N (2022). Phenotypic characterization of gamma delta (gammadelta) T cells in chickens infected with or vaccinated against Marek’s disease virus. Virology.

[17] Hernandez R, Brown DT (2010). Growth and maintenance of chick embryo fibroblasts (CEF). Curr Protoc Microbiol.

[18] Schat KA, Calnek BW, Fabricant J (1982). Characterisation of two highly oncogenic strains of Marek’s disease virus. Avian Pathol.

[19] von Heyl T, Klinger R, Aumann D, Zenner C, Alhussien M (2023). Loss of alphabeta but not gammadelta T cells in chickens causes a severe phenotype. Eur J Immunol.

[20] Bello N, Francino O, Sánchez A (2001). Isolation of genomic DNA from feathers. J Vet Diagn Invest.

[21] Conradie AM, Bertzbach LD, Trimpert J, Patria JN, Murata S (2020). Distinct polymorphisms in a single herpesvirus gene are capable of enhancing virulence and mediating vaccinal resistance. PLoS Pathog.

[22] Jarosinski K, Kattenhorn L, Kaufer B, Ploegh H, Osterrieder N (2007). A herpesvirus ubiquitin-specific protease is critical for efficient T cell lymphoma formation. Proc Natl Acad Sci USA.

[23] Jarosinski KW, Margulis NG, Kamil JP, Spatz SJ, Nair VK (2007). Horizontal transmission of Marek’s disease virus requires US2, the UL13 protein kinase, and gC. J Virol.

[24] Kaufer BB, Jarosinski KW, Osterrieder N (2011). Herpesvirus telomeric repeats facilitate genomic integration into host telomeres and mobilization of viral DNA during reactivation. J Exp Med.

[25] Seliger C, Schaerer B, Kohn M, Pendl H, Weigend S (2012). A rapid high-precision flow cytometry based technique for total white blood cell counting in chickens. Vet Immunol Immunopathol.

[26] Matsuyama-Kato A, Shojadoost B, Boodhoo N, Raj S, Alizadeh M (2023). Activated chicken gamma delta T cells are involved in protective immunity against Marek’s disease. Viruses.

[27] Djeraba A, Musset E, Lowenthal JW, Boyle DB, Chaussé A-M (2002). Protective effect of avian myelomonocytic growth factor in infection with Marek’s disease virus. J Virol.

[28] Fakhrul Islam AF, Walkden-Brown SW, Groves PJ, Underwood GJ (2008). Kinetics of Marek’s disease virus (MDV) infection in broiler chickens 1: effect of varying vaccination to challenge interval on vaccinal protection and load of MDV and herpesvirus of turkey in the spleen and feather dander over time. Avian Pathol.

