# Supplementary material for: Role of γδ T cells in turkey herpesvirus vaccine protection against Marek’s disease virus
Source: J Gen Virol. 2026 Jan 30;107(1):002204. doi: 10.1099/jgv.0.002204 (PMC12859218; doi:10.1099/jgv.0.002204)
Supplement: Uncited Fig. S1. [file jgv-107-02204-s001.pdf]

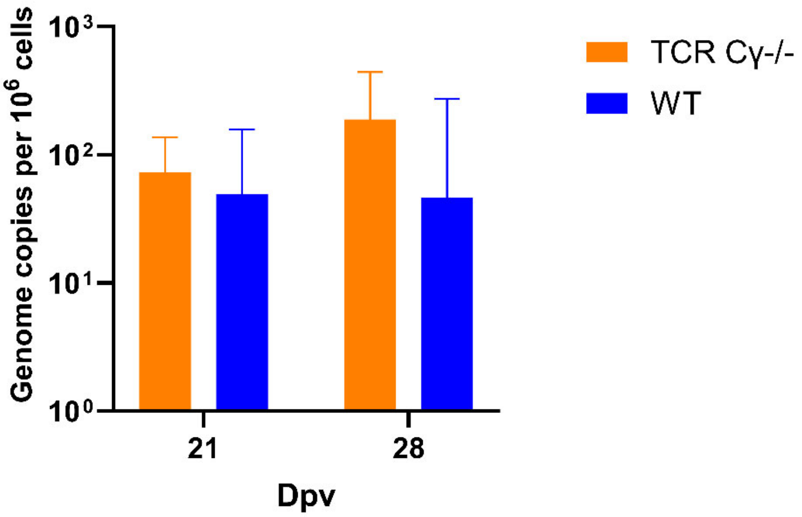

Supplementary Figure 1. qPCR analysis of virus load in the FFE. HVT genomes in feather tip material from both groups (n = 8 per group) were quantified by qPCR targeting the SORF1 gene. Data is shown as means ± standard deviations (*p ≤ 0.05, Mann–Whitney U test).
